# Vaccine Protection of Mice With Primary Immunodeficiencies Against Disseminated Coccidioidomycosis

**DOI:** 10.3389/fcimb.2021.790488

**Published:** 2022-01-07

**Authors:** Daniel A. Powell, Amy P. Hsu, Christine D. Butkiewicz, Hien T. Trinh, Jeffrey A. Frelinger, Steven M. Holland, John N. Galgiani, Lisa F. Shubitz

**Affiliations:** ^1^ Valley Fever Center for Excellence, University of Arizona, Tucson, AZ, United States; ^2^ Department of Immunobiology, University of Arizona, Tucson, AZ, United States; ^3^ Laboratory of Clinical and Infectious Diseases, National Institutes of Allergy and Infectious Disease, Bethesda, MD, United States; ^4^ Department of Medicine, University of Arizona, Tucson, AZ, United States

**Keywords:** coccidioidomycosis, vaccine, disseminated, immunodeficiency, mice

## Abstract

Disseminated coccidioidomycosis (DCM), often a severe and refractory disease leading to poor outcomes, is a risk for people with certain primary immunodeficiencies (PID). Several DCM-associated PID (STAT4, STAT3, IFNγ, and Dectin-1) are modeled in mice. To determine if vaccination could provide these mice protection, mice with mutations in *Stat4*, *Stat3*, *Ifngr1*, *Clec7a* (Dectin-1), and Rag-1 (T- and B-cell deficient) knockout (KO) mice were vaccinated with the live, avirulent, Δ*cps1* vaccine strain and subsequently challenged intranasally with pathogenic *Coccidioides posadasii* Silveira strain. Two weeks post-infection, vaccinated mice of all strains except Rag-1 KO had significantly reduced lung and spleen fungal burdens (p<0.05) compared to unvaccinated control mice. Splenic dissemination was prevented in most vaccinated immunodeficient mice while all unvaccinated B6 mice and the Rag-1 KO mice displayed disseminated disease. The mitigation of DCM by Δ*cps1* vaccination in these mice suggests that it could also benefit humans with immunogenetic risks of severe disease.

## Introduction

Coccidioidomycosis is a systemic fungal infection of the American southwest caused by the endemic fungi, *Coccidioides immitis* and *C. posadasii*. They are primary pathogens which cause disease in immunologically normal hosts (Nguyen et al., 2013). The consequences of infection, typically acquired by inhaling fungal spores in soil and air, range from asymptomatic in about 60% of people to a wide spectrum of clinical illness in the remainder (Drutz and Catanzaro, 1978; Nguyen et al., 2013). The majority of clinical cases are uncomplicated pneumonia and resolve without treatment over a period of weeks to months (Galgiani et al., 2019). Approximately 1% of all infections result in progressive disease beyond the chest, a complication known as disseminated coccidioidomycosis (DCM) (Borchers and Gershwin, 2010; Odio et al., 2017). Established risk factors for DCM include AIDS, pregnancy, race/ethnicity, and exogenous immunosuppression (steroids, antirejection drugs, biological immunosuppressants, antineoplastic drugs) (Nguyen et al., 2013; Odio et al., 2017). People with rare primary immunodeficiencies (PID) in a variety of signaling pathways, including the IL-12/IFNγ axis and STAT3 pathway, have also been identified with disseminated coccidioidomycosis (DCM) and the mutations are thought to make them more susceptible (Mansouri et al., 2005; Odio et al., 2017; Hung et al., 2019; Powell et al., 2019).

The IL-12/IFNγ axis is critical for development of adaptive immune responses and for killing of intracellular bacteria, such as mycobacteria and *Salmonella*, as well as fungal pathogens (Mansouri et al., 2005; Odio et al., 2017). Engagement of pattern recognition receptors, including the β-glucan receptor Dectin-1, on antigen presenting cells induces the release of IL-12, leading to activation of T- and NK-cells through STAT family transcription factors and cytokine production, including IL-23 and IFNγ. IFNγ then drives the release of microbicidal factors from macrophages (Hung et al., 2019). Immunodeficiencies in these pathways result in the inability to mount an appropriate adaptive response, resulting in weak or absent killing of microbes. We wondered whether it might be possible to mitigate the effects of primary immunodeficiencies on disseminated coccidioidomycosis through vaccination. Mouse models of vaccination and challenge were used to explore this question.

Removal of the 6 kb *CPS1* gene from *C. posadasii*, strain Silveira, resulted in the avirulent strain, Δ*cps1*. When used as a vaccine, Δ*cps1* extends survival and greatly diminishes dissemination in susceptible but immunologically normal mice (Narra et al., 2016; Shubitz et al., 2018). C57BL/6 mice infected with 50-100 spores of Silveira typically develop total lung fungal burdens >1 x 10^6^ colony-forming units (CFU) by day 14 post-infection; when tested for survival, all of them die in less than three weeks (Narra et al., 2016; Shubitz et al., 2018). By contrast, vaccinated mice have mean total lung fungal burdens <1 x 10^2^ CFU, splenic dissemination cannot be detected grossly or by fungal culture in the majority of animals, and all mice survived six months after challenge with either *C. immitis* or *C. posadasii* (Shubitz et al., 2018). Given the success of Δ*cps1* vaccination in normal, susceptible mice, we surveyed PID mouse models of vaccination and challenge to test its effectiveness.

## Materials and Methods

### Mice

Commercially available mice were purchased from Jackson Laboratories (Bar Harbor, Maine): C57BL/6J (B6, stock #000664), B6D2F1/J (stock #10006), Stat4 KO (C57BL/6J-*Stat4^em3Adiuj^* ^/^J, stock #028526), and Ifngr KO (B6.129S7-*Ifngr1^tm1Agt^*/J stock # 003288) (Huang et al., 1993). Dominant-negative mutant Stat3 (mut-Stat3) that models hyperimmunoglobulin E syndrome (C57BL/6-Tg(Stat3*)9199Alau/J, stock #027952) (Steward-Tharp et al., 2014) and Dectin-1 KO mice (B6.129S6-*Clec7a^tm1Gdb^*/J, stock #012337) (Marakalala et al., 2013) were purchased from Jackson Laboratories and bred in house to obtain sufficient numbers. Stat4^E626G^ (B6-*Stat4^em1Doe/em1Doe^*) mice, recapitulating a mutation in three generations of a family with disseminated coccidioidomycosis (Powell et al., 2019), were engineered using CRISPR/Cas9 and bred in house (Singh et al., 2015; Powell et al., 2020). Stat4^E626G/E626G^ females were crossed with DBA2/J males to obtain heterozygous, dominant-negative B6D2F1 offspring. Rag-1 KO mice (B6.129S7-*Rag1^tm1Mom^*/J) were a generous gift of J. Nikolich-Zugich and were used as T- and B-cell deficient controls unable to mount an adaptive immune response. Both male and female mice were used in all studies. Breeding of mice and procedures were approved by the institutional animal care and use committee for the University of Arizona. Mice were housed and cared for according to PHS standards, and all manipulation of infected mice was performed at animal biosafety level (BSL) 3.

### Fungal Cultures

*C. posadasii* strain Silveira (Silveira) (ATCC #28868), a highly pathogenic strain, and the avirulent vaccine strain, Δ*cps1*, (Narra et al., 2016) were grown on 2x glucose-yeast extract agar (2% glucose, 1% yeast extract, 1.5% agar) at 30°C until colonies appeared mature as previously described (Narra et al., 2016). In brief, arthroconidia were harvested by the spin-bar method in water, enumerated by hemocytometer, and cultured to determine viability by plating serial dilutions and counting CFU at 72 hrs (Silveira) or 96 hrs (Δ*cps1*). Prior to intranasal infection or vaccine inoculation, arthroconidia were diluted to the desired concentration in sterile saline for injection. To determine fungal burdens post-sacrifice, organs were homogenized in 1 mL of sterile, isotonic saline. Ten-fold serial dilutions (100 µl/plate), plus residual volume of undiluted homogenate from mice with minimal or no gross disease, were incubated for 72 hrs and organ CFU determined from plate colony counts. All growth and manipulation of *Coccidioides* fungi was performed at BSL3 for Silveira and BSL2 or BSL3 for Δ*cps1* vaccine strain. The avirulent Δ*cps1* strain is approved by the Institutional Biosafety Committee of the University of Arizona for use at BSL2.

### Vaccine Challenge Studies

Six to 12 week old mice in groups of 7-10 animals were vaccinated twice subcutaneously with approximately 10,000 viable arthroconidia (range 7800-14,650) of *Δcps1* in the right groin followed by the left groin two weeks later. Negative control mice received two injections of isotonic saline. Four weeks after booster, mice were infected intranasally under ketamine (80 mg/kg)-xylazine (8 mg/kg) anesthesia with 50-100 arthroconidia of Silveira administered in 30 µl of sterile saline dropwise into the nares with a micropipettor. Infectious doses were verified by plate culture of the inoculum following infection of the mice. Starting day 7 p.i., mice were monitored daily for weight, activity level, and hydration. Animals exhibiting dehydration, hunched posture, isolation from cagemates, weight loss >25%, and lethargy or weakness were euthanized as needed prior to planned end of study. Studies were terminated on day 14 post-infection and lungs and spleens collected for quantitative fungal culture.

### Statistical Analysis

Raw organ fungal burden data were log transformed. Zero was replaced with 1.04 and 1 CFU was replaced with 2 prior to log transformation and plotting. Log-transformed data were tested for normality. Data were subsequently analyzed by the nonparametric Kruskal-Wallis test with a Dunn’s test for multiple comparisons. Differences were considered significant at p ≤ 0.05.

## Results

### Vaccination Reduces Fungal Burden in PID Mouse Strains

**Figure 1:**
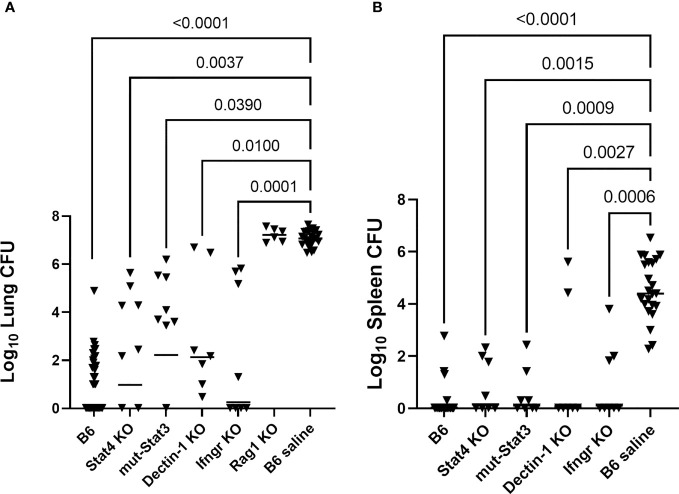
Mice with primary immunodeficiencies and normal C57BL/6 (B6) mice were vaccinated twice with Δ*cps1* avirulent vaccine and challenged IN with 50-100 arthroconidia of virulent *C. posadasii* Silveira. **(A)** Lung fungal burdens quantitated 14 days post-infection were significantly reduced in Stat4 KO, mut-Stat3, Dectin-1 KO and Ifngr KO mice compared to unvaccinated B6. Vaccinated Rag-1 KO mice, deficient in T- and B-cells, had no reduction in fungal burdens. **(B)** Dissemination was prevented in most vaccinated PID mice and statistically significantly reduced in all strains while universally present in unvaccinated B6 mice. (Statistical analysis – Kruskal- Wallis).

One vaccine challenge study was performed for each of the following strains of mice with a primary immunodeficiency: Stat4 KO, Ifngr KO, mut-Stat3, Dectin-1 KO, Rag-1 KO. B6 mice were used for vaccinated and unvaccinated controls. B6 controls from four of the studies, which bracketed the low and high infection doses (range, 50-99 arthroconidia), were combined to use as controls for statistical analysis. **Figure 1** shows the composite results of the lung and spleen fungal burdens of vaccinated PID mice compared with B6 vaccinated and saline control mice. There were significant reductions in lung fungal burdens of the Stat4 KO, mut-Stat3, Ifngr KO, and Dectin-1 KO mice compared to the unvaccinated B6 mice (P<0.05, all comparisons, Kruskal-Wallis). As expected based on the known requirement for T-cells in coccidioidal immunity (Cox and Magee, 2004; Fierer et al., 2006), vaccination of Rag-1 KO mice failed to reduce fungal burden (P>0.99 compared to unvaccinated B6).

Spleen fungal burdens were undetectable in 58% of the vaccinated PID mice and 88% of the vaccinated B6 mice. In contrast, splenic dissemination was universal in the unvaccinated B6 mice. Spleen fungal burdens for each strain were significantly reduced compared to unvaccinated B6 mice (P ≤ 0.003, all comparisons). (**Figure 1B**) Spleen fungal burdens were not quantitated for the Rag-1 KO mice, but all spleens and livers from those mice were positive for fungal growth, demonstrating dissemination similar to the unvaccinated mice. Overall, vaccination of Stat4 KO, mut-Stat3, Ifngr KO, and Dectin-1 KO mice reduced lung burdens and diminished dissemination.

### Vaccinated B6D2F1-*Stat4* ^E626G/+^ Are Indistinguishable From B6D2F1 Mice

**Figure 2:**
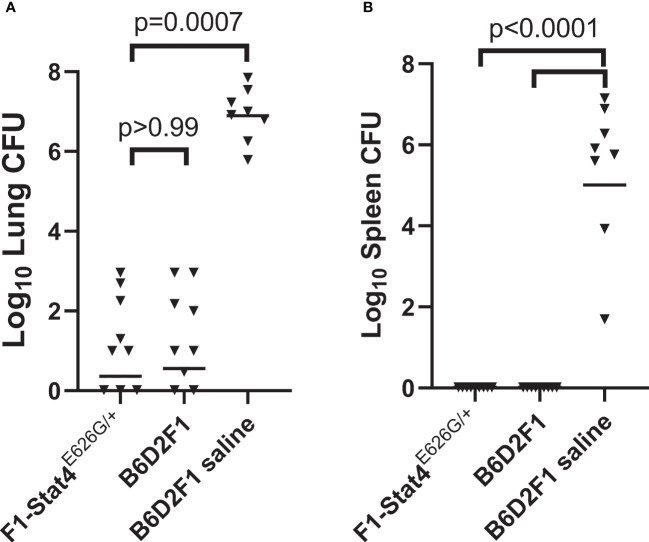
B6D2F1-Stat4^E626G/+^ mice vaccinated with Δ*cps1* twice and challenged with lethal *C. posadasii* Silveira had significantly reduced lung **(A)** and spleen **(B)** fungal burdens 14 days post-infection. They were indistinguishable from normal B6D2F1 mice. Dissemination was prevented in B6D2F1-Stat4^E626G/+^ mice while universally present in unvaccinated B6D2F1 mice. (Statistical analysis, Kruskal-Wallis).

B6-Stat4^E626G/E626G^ mice were bred to DBA/2J mice to create heterozygous mice with a dominant-negative mutation present in three generations of family members with disseminated coccidioidomycosis. For background, *Coccidioides* infection with Silveira is so lethal to B6 mice that there was significant concern negative effects of the mutation would not be observed in experiments with B6-Stat4^E626G/E626G^ or B6-Stat4^E626G-/+^ mice. DBA/2 and B6D2F1 mice are both more resistant to coccidioidomycosis (Cox et al., 1988; Fierer et al., 1999; Shubitz et al., 2021), so the cross to the genetically more resistant DBA/2 mice was bred to test the negative impact of the allele. Purchased B6D2F1/J mice were used as vaccinated and unvaccinated controls. **Figure 2** shows that vaccination of the B6D2F1-Stat4*^E626G-/+^* mice resulted in very low lung fungal burdens indistinguishable from the vaccinated B6D2F1 mice (P>0.99). Vaccination entirely prevented dissemination to the spleen as it did in the vaccinated B6D2F1 mice, while both lung and spleen fungal burdens were high in the unvaccinated animals.

## Discussion

This series of vaccination-challenge studies in mice with PID homologous to humans with DCM demonstrates that the avirulent Δ*cps1* vaccine induced some protection in all of them. Reduction in dissemination was one of the most remarkable outcomes of vaccination in these mice. In the case of the Stat4 mutation from a family with 3 generations of disseminated coccidioidomycosis, the vaccinated mice showed no dissemination and appeared as protected as normal mice in this short term study. Therefore, it seems plausible that Δ*cps1* vaccine may protect vulnerable patients with PID from severe, progressive disease. At minimum, a reduction in disease severity from vaccination might improve outcomes of antifungal treatment. Some DCM patients identified with PID, including the family with the *Stat4* ^E626G^ mutation, had no previous medical histories before developing severe DCM (Odio et al., 2017; Powell et al., 2019). This speaks to the concept that there may be many unidentified carriers of negative alleles who would potentially avoid the most severe outcomes of coccidioidomycosis through vaccination.

Hung, *et.al*., showed that vaccine immunity is driven by early activation of Th1, Th2, and Th17 pathways using the live, attenuated *Coccidioides* vaccine, ΔT (Hung et al., 2011). Consistent with this, Rag-1 KO mice, lacking all mature lymphocyte lineages, were not protected by Δ*cps1* vaccine. It also demonstrates that vaccine protection from Δ*cps1* is not a result of trained innate immunity, since the Rag-1 KO mice have an intact myeloid compartment (Blok et al., 2015). Hung et al., further showed that IL-17ra KO^-/-^ mice have poor adaptive immunity from failure to generate Th17 cells. Interestingly, we showed partial vaccine protection in a mouse model of hyper-immunoglobulin E syndrome (HIES), an immunodeficiency mainly associated with susceptibility to *Staphylococcus aureus*, recurrent pneumonia, and *Candida albicans*, but for which there are reports of disseminated coccidioidomycosis (Powers et al., 2009; Odio et al., 2015). Humans with HIES have heterozygous, dominant-negative mutations in *STAT3* and circulating Th17 cells are absent or low (Renner et al., 2008). In light of varying results of vaccination challenge studies, additional studies on mice with Stat3/Th17 abnormalities are needed to better predict the utility of vaccination in this particular population.

Use of Ifngr KO mice demonstrated IFNγ signaling is not required for a *Coccidioides* vaccine response in mice administered ΔT, another live, attenuated coccidioidal vaccine strain of *C. posadasii* (Fierer et al., 2006; Hung et al., 2011). This is consistent with our study results for the avirulent Δ*cps1* vaccine. Even though defects in IL-12 and IFNγ receptors are associated with severe, disseminated coccidioidomycosis and non-tuberculous mycobacterial infections in humans, (Dorman and Holland, 1998; Dorman et al., 2004; Vinh et al., 2009; Odio et al., 2017), murine models suggest that vaccination might provide significant protection to this susceptible population.

Dectin-1 has also been shown to be very important in driving adaptive immunity to *Coccidioides* through IFNγ and IL-17, affecting both Th1 and Th17 pathways (Hung et al., 2019). Damaging Dectin-1 variants are overrepresented in a cohort of human patients with disseminated coccidioidomycosis (Hsu et al., 2020). However, vaccination reduced fungal burden and dissemination in Dectin-1 KO mice in our experiment. This suggests additional receptors involved in *Coccidioides* recognition allow a sufficiently effective vaccine response that diminishes the severity of infection. More studies are needed to verify and understand this.

Limitations of this data include the lack of confirmatory studies of protection and mechanistic studies to understand how Δ*cps1* stimulates immunity with defects in the primary pathways leading to adaptive immunity in fungal disease. Survival studies and determination of the pathways Δ*cps1* antigens use to generate an immune response are important for follow up on this preliminary work. The data generated here suggest there is a range of protection; additional studies would also identify which immunological defects are the most deleterious and difficult to protect.

To summarize, Δ*cps1* vaccination provided reduction of disseminated disease in mouse strains that model human primary immunodeficiencies associated with severe DCM. Additional studies should focus on the role of vaccines to prevent or mitigate disease in people with mutations or alleles which make them susceptible to DCM.

## Data Availability Statement

The raw data supporting the conclusions of this article will be made available by the authors, without undue reservation.

## Ethics Statement

The animal study was reviewed and approved by University of Arizona Institutional Animal Care and Use Committee.

## Author Contributions

DP, AH, JF, SH, JG, and LS designed the experiments. DP, AH, CB, HT, and LS carried out experiments, collected and analyzed data. LS wrote the original draft of the manuscript. All authors participated in the editing of the manuscript. All authors contributed to the article and approved the submitted version.

## Funding

This work was supported by grants from the National Institute of Allergy and Infectious Disease R01AI132140 to JNG and U01AI122275 to SH and JG.

## Conflict of Interest

The authors declare that the research was conducted in the absence of any commercial or financial relationships that could be construed as a potential conflict of interest.

## Publisher’s Note

All claims expressed in this article are solely those of the authors and do not necessarily represent those of their affiliated organizations, or those of the publisher, the editors and the reviewers. Any product that may be evaluated in this article, or claim that may be made by its manufacturer, is not guaranteed or endorsed by the publisher.
